# Enhanced Environmental Hydroxylamine Detection: A Chromatographic Adaptation to the Indooxine Approach

**DOI:** 10.1021/acsomega.6c04322

**Published:** 2026-07-08

**Authors:** Madelyn Y. Hathcock Barden, Kevin M. McPeak, Samuel D. Snow

**Affiliations:** † Department of Civil and Environmental Engineering, 5779 Louisiana State University, 3255 Patrick Taylor Hall, Baton Rouge, Louisiana 70803, United States; ‡ Gordon and Mary Cain Department of Chemical Engineering, 5779 Louisiana State University, 3307 Patrick Taylor Hall, Baton Rouge, Louisiana 70803, United States

## Abstract

Hydroxylamine (NH_2_OH) plays an important role as an intermediate species in many nitrogen transformation reactions. Since it readily reacts with O_2_, NH_2_OH is often present in submicromolar concentrations in environmental systems. Existing NH_2_OH quantification protocols are limited by sensitivity, cost, or both; consequently, the extent and role of NH_2_OH in many biological and abiotic processes are poorly understood. In the work presented here, a colorimetric method is improved and adapted for use in high-performance liquid chromatography (HPLC). The method entails the reaction of NH_2_OH with a reagent, 8-hydroxyquinoline, to form a larger compound, indooxine, that absorbs strongly in the visible range at 705 nm and at 261 nm in the UV window. Using a HPLC-UV system detecting at the 261 nm peak provided a significantly enhanced NH_2_OH detection limit and improved measurement reliability. This chromatographic method was tested for sensitivity and performance in wastewater and river water matrices, in addition to pure water cases, achieving reliable results in each solution. The protocol achieved accurate measurements of NH_2_OH concentrations as low as 0.05 μM, vastly improving previous detection limits. Sampling procedures that ensure the preservation of NH_2_OH were also established; samples acidified below a pH of 2.0 remained stable under room temperature conditions for up to two months. Given that HPLC-UV systems are relatively common in environmental laboratories, the adapted method reported here is expected to significantly improve the accessibility of accurate, highly sensitive NH_2_OH detection.

## Graphical abstract

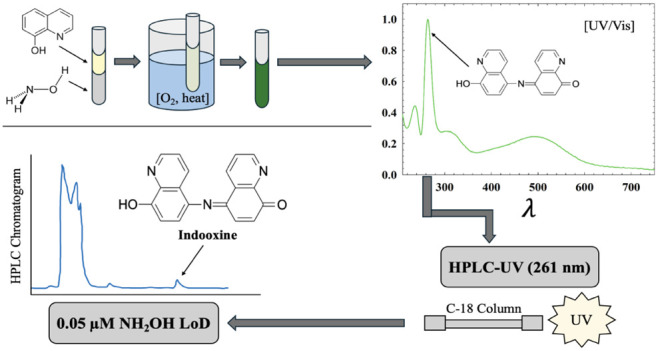

## Introduction

With the growing interest in hydroxylamine’s (NH_2_OH) role in the nitrogen cycle,^1^ as well as its role in the production of nitrous oxide gas (N_2_O) in wastewaters,^2^ there is a strong motive to study NH_2_OH in natural and engineered biological systems. NH_2_OH is an unstable compound within natural aqueous settings,^3^ and accurate methods for NH_2_OH quantification have limited sensitivity.^4^

Several approaches have been used to quantify NH_2_OH, such as the use of electrodes,^5–9^ spectrophotometry,^10^ and gas chromatography,^11^ among others.^12,13^ Previous NH_2_OH quantification methods and their corresponding limit of detections (LoD) are presented in Table 1. In 2004, Seike et al. reported a method of determining NH_2_OH levels in fresh-water samples by oxidizing NH_2_OH into N_2_O using sodium hypochlorite.^14^ Their method used gas chromatography coupled with an electron-capture detector (GC-ECD) to measure N_2_O, such that NH_2_OH was quantified by the 1:2 molar conversion of NH_2_OH to N_2_O in the samples. Seike et al. observed that their method has limitations and cannot be applied for use within brackish water. They reported a detection limit of 0.1 μgN/L, or 0.00357 μM N_2_O, which corresponds to a NH_2_OH detection limit of 0.00714 μM. In 2017, Kato et al. adapted Seike et al.’s methods to be applicable for use in brackish and seawater samples by using the addition of a phenol solution to overcome interfering reactions of bromide with the chlorine.^15^ Kato et al. reported a detection limit of 0.2 μgN/L, which corresponds to a detection limit of 0.0143 μM NH_2_OH. While these reports provide a highly sensitive method for NH_2_OH analysis, several limitations remain: sample preparation and handling require careful control of solution volume to avoid headspace, and GC-ECD systems are expensive and uncommon in environmental laboratories.

**Table 1:** Environmental NH_2_OH Detection Techniques and Their Limitations

Detection Technique	Reported LoD	Equipment/Materials Required	Limitations and Interferences
Indooxine colorimetry with HPLC-UV (proposed method)	0.05 μM	HPLC-UV	Precipitation interference (sometimes requires dilution)
Indooxine colorimetry, Frear and Burrell, 1955	10 μM	Spectrophotometer	Low sensitivity
Spectrophotometry, Hu et al., 2018	1.51 μM	Spectrophotometer	Low sensitivity, interference by NO_2_^–^ > 15 mg/L
Ratiometric fluorescent probe, Du et al., 2024	0.19 μM	Phenothiazine-based ratiometric fluorescent probe (PCHO)	Custom-synthesized fluorescent probe required
N_2_O conversion, Seike et al., 2004	0.00714 μM	GC-ECD	Interference by Br^–^, tedious, cost
N_2_O conversion, Kato et al., 2017	0.0143 μM	GC-ECD	Tedious, cost
Modified glassy carbon electrode (GCE), Zhang and Zheng, 2012	0.1 μM	GCE amended with custom-synthesized nanoparticles	Custom-synthesized materials required, interference by NO_2_^–^, S_2_O_3_^2–^, I^–^, and hydrazine at 100 × [NH_2_OH]
Modified carbon paste electrode (CPE), Ensafi et al., 2013	0.15 μM	CPE amended with custom-synthesized nanoparticles	Custom-synthesized materials required
Modified CPE, Sadeghi et al., 2013	0.08 μM	CPE amended with custom-synthesized nanoparticles	Custom-synthesized materials required
Biamperometry, Zhao and Song, 2001	0.1 μM	Flow-injection biamperometry	Highly specific instrumentation required, interference by hydrazine and hydrochloride at 0.1 × [NH_2_OH] and NH_4_^+^, Ag^+^, and several organic acids at 10 × [NH_2_OH]

Another method for detecting NH_2_OH within natural waters is the production of indooxine through a reaction involving NH_2_OH. Frear and Burrell reported a method for detecting NH_2_OH using a spectrophotometer in 1955.^16^ Their procedure is an adaptation of a method reported by Berg and Becker in 1940 that involves a reaction between NH_2_OH and 8-hydroxyquinoline to form a stable compound of 5,8-quinolinequinoe-5-(8-hydroxy-5-quinolylimide), called indooxine,^17^ as shown in Scheme 1.^18^ This colorimetric method is not nearly as sensitive as the GC-ECD; Frear and Burrell reported that the method could only accurately detect NH_2_OH concentrations down to roughly 10 μM NH_2_OH.

**Scheme 1:**
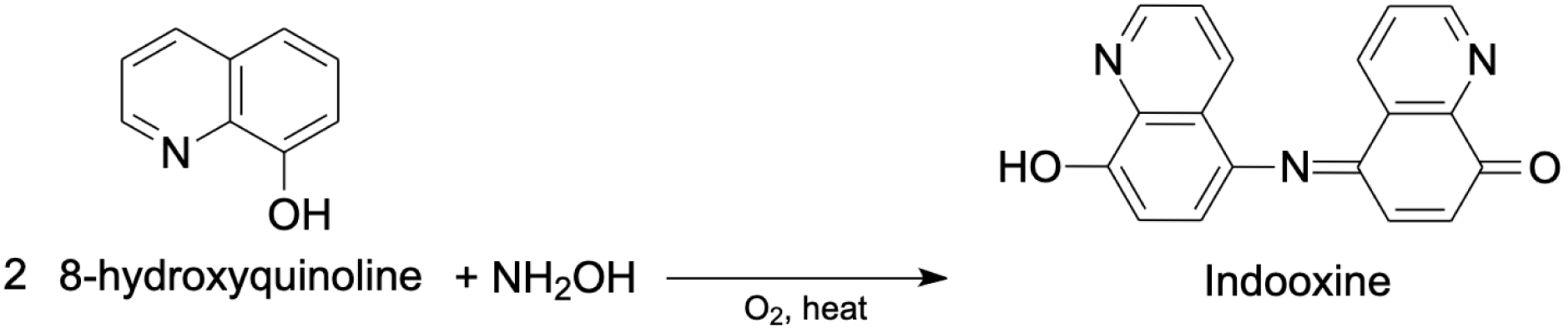
Schematic of the Reaction of NH_2_OH with 8-Hydroxyquinoline in the Presence of Oxygen to Form Indooxine

With an interest in quantifying NH_2_OH at lower concentrations and creating a widely accessible detection platform, the following method was developed, which adapts Frear and Burrell’s indooxine colorimetric method for use in a high-pressure/performance liquid chromatography (HPLC) system with a UV detector for enhanced measurement accuracy. The HPLC-UV system enables a significant lowering of the NH_2_OH LoD, utilizing a common apparatus found in most environmental laboratories. The procedures reagents are affordable, so costs are approximated by the cost of HPLC operation, and each sample requires about 20 min to perform. This method was applied to wastewater, brackish water, and freshwater samples. Best practices for sample collection and handling for the unstable NH_2_OH compound were determined by testing NH_2_OH decay under different conditions. The current relevant environmental NH_2_OH detection techniques discussed in this paper, as well as their respective limitations are summarized in Table 1. Others have described precise NH_2_OH identification methods, such as an NH_2_OH-oxime reaction followed by 1H NMR,^19^ but this NMR approach is unrealistic for environmental analysis with a LoD in the mM range, sophisticated instrumentation requirements, and a 24 h reaction time frame prior to analysis.

## Experimental Section

### Materials and Chemicals

Hydroxylamine hydrochloride (NH_2_OH.HCl), sodium phosphate monobasic anhydrous (Na_2_HPO_4_), sodium phosphate dibasic (Na_2_HPO_4_.7H_2_O), trichloroacetic acid (C_2_HCl_3_O_2_), hydrochloric acid 6.0 N (HCl), sulfuric acid 6.0 N (H_2_SO_4_), sodium hydroxide 1.0 N (NaOH), a sodium hypochlorite solution (5% available chlorine, NaOCl), phosphoric acid (85% ACS grade), and HPLC-grade methanol were purchased from VWR (Radnor, PA). 8-Hydroxyquinoline, 99% (8-quinolinol), was obtained from Beantown Chemical (Hudson, NI). Sodium carbonate, 99+% (Na_2_CO_3_) was purchased from Acros Organics (Geel, Belgium). HPLC grade acetonitrile was purchased from Sigma-Aldrich (Darmstadt, Germany). Ultrapure water was used for all reagent preparations except for 8-hydroxyquinoline. Reagent solutions were prepared as follows:

Hydroxylamine: A 100 mM NH_2_OH working stock solution was prepared by dissolving NH_2_OH.HCl crystals in 100 mL of water. The NH_2_OH working stock was adjusted to a pH below 2.0 using HCl for stability. Fresh dilutions of this stock solution were prepared for different NH_2_OH concentration solutions at the time of use.

Phosphate Buffer: A 0.4 M phosphate buffer solution was prepared by dissolving Na_2_HPO_4_ in 100 mL of water. The phosphate solution was then buffered to a pH of 6.8 using a 0.4 M Na_2_HPO_4_.7H_2_O solution, which was prepared by dissolving Na_2_HPO_4_.7H_2_O in 100 mL of water.

Trichloroacetic Acid: A 1.197 M trichloroacetic acid solution, 12% by weight, was prepared by dissolving C_2_HCl_3_O_2_ in 100 mL of water.

8-Hydroxyquinoline: A 0.3 M 8-hydroxyquinoline solution was prepared by adding 8-hydroxyquinoline (99%) to 100 mL of 80% biotechnology grade ethanol, which dissolved rapidly with gentle mixing.

Sodium Carbonate: A 1.0 M sodium carbonate solution was prepared by dissolving Na_2_CO_3_ in 100 mL of water.

### Sample Collection Procedure

The wastewater samples used in the following experiments were collected at the overflow of the South Baton Rouge Wastewater Treatment Plant (SBRWWTP) clarifiers, immediately prior to the disinfection contactor. Freshwater samples were collected from the shorelines of the Mississippi River and the Louisiana State University (LSU) Lakes in Baton Rouge, Louisiana. Previous literature used H_2_SO_4_ as the acidifying compound, which was applied to the collection of natural water samples.^15^ All samples were acidified by adding 0.6 N H_2_SO_4_ to the sample (2% by volume) to achieve a pH below 2.0 and stabilize NH_2_OH on site before bringing back to the lab for testing. Acidified samples were refrigerated at 5 °C within 30 min of collection and stored until the time of use. All samples were filtered using VWR 0.45 μm hydrophilic PTFE syringe filters (Radnor, PA) prior to being used within the quantification procedures.

### Hydroxylamine Quantification Procedure

NH_2_OH was quantified by first inducing the formation of indooxine as a product of the reaction between NH_2_OH and 8-hydroxyquinoline; the indooxine was then measured according to the procedures described below. The reaction solutions were prepared according to Table 2 below; additional information regarding stock concentrations and volumes used is provided in Table S1 of the Supporting Information (SI).

**Table 2:** Reagent Concentrations within the Solution Used to Form Indooxine

Reagent	Concentration in Final Solution (M)
Phosphate buffer (6.8 pH)	0.01
Trichloroacetic acid	0.03
8-hydroxyquinoline	0.0075
Sodium carbonate	0.125

After the addition of the phosphate buffer, C_2_HCl_3_O_2_, and 8-hydroxyquinoline, the solutions were shaken gently before adding sodium carbonate and shaken vigorously to ensure complete oxidation by atmospheric O_2_.^16^ A blank solution was prepared in tandem, substituting the NH_2_OH-containing sample with ultrapure water. The solutions were then placed within a hot water bath at boiling point for 1 min. Attempts to use a lower temperature (60 °C) with a longer reaction time (10 min) were less accurate and not pursued further. After heating, the yellow color from the 8-hydroxyquinoline changes to green with the formation of the blue indooxine dye when NH_2_OH is present. The solutions were removed from the hot water bath and cooled for 15 min at room temperature before analysis. Samples were measured within 1 h of cooling to avoid significant decay of indooxine (decay was observed after about 2 h, as shown in Table S2).

Experimental data sets were collected on multiple instruments, which allowed for thorough analysis regarding the absorbance and detection limit of NH_2_OH. A VWR (Radnor, PA) UV-3100PC spectrophotometer was used to measure the absorbance of indooxine within a high detection range; the peak indooxine absorbance was measured at a 705 nm wavelength following Frear and Burrell’s colorimetric method.^16^ An Avantes (Apeldoorn, Netherlands) AvaSpec-ULS2048XL-EVO spectrophotometer coupled with an LS-1 tungsten halogen lamp, Avantes fiber cables, and a 10 cm long-pass optical cell was able to improve the colorimetric detection limit.

To further improve sensitivity, a method was developed to measure the absorbance of indooxine using an HPLC system coupled with a UV detector. An Agilent Technologies 1260 Infinity was used here, with an ACE Equivalence 3 C18 column (125 × 3.0 mm i.d.; United Kingdom), which was purchased from VWR (Radnor, PA) to separate the indooxine compound from other molecules within the sample solution. The HPLC’s UV detector was set to detect at a 261 nm peak wavelength to measure the absorbance of indooxine within the sample. An injection volume of 100 μL was used with a flow rate of 0.5 mL per minute. The mobile phase was comprised of 10% acetonitrile and 90% 10 mM phosphoric acid solution until 5 min to elute the 8-hydroxyquinoline and other constituents while retaining the indooxine. After 5 min, the solvent ratio was adjusted to a mixture of 10% acetonitrile, 10% methanol, and 80% 10 mM phosphoric acid solution for the remaining 5–12 min, which eluted indooxine from the column. The indooxine peak was observed between 7 and 10 min, as shown in Figure S1.

## Results and Discussion

### Adaptation of Previous 8-Hydroxyquinoline/Indooxine Methods

Frear and Burrell’s colorimetric method calls for a solution consisting of only 20% of the NH_2_OH-containing sample, with the remaining 80% of the volume occupied by reagents; given this limitation, they only reported down to 10 μM of NH_2_OH.^16^ To achieve a lower detection limit, the modifications here adapted Frear and Burrell’s recipe to consist 80% of the NH_2_OH-containing sample and 20% reagents (Table 2). The revised recipe’s reagent concentrations, final solution concentrations, and volume ratios were slightly altered from Frear and Burrell’s original recipe to overcome solubility issues with 8-hydroxyquinoline and sodium carbonate, as shown in Table S1.

The revised recipe was validated with high concentration samples using the 705 nm colorimetric detection, measuring the absorbance of indooxine. However, even with a long-pass cell, the spectrophotometric method was unable to detect low range NH_2_OH concentrations (<2.5 μM). The isolation of the indooxine molecule from 8-hydroxyquinoline and other reagents using the HPLC allowed for the use of indooxine’s stronger absorbance at UV wavelengths. The UV/vis absorption spectra were collected to determine an alternative peak absorbance within the UV range for indooxine; the peak absorbance was found to be detected around 261 nm, as shown in Figure 1.

**Figure 1:**
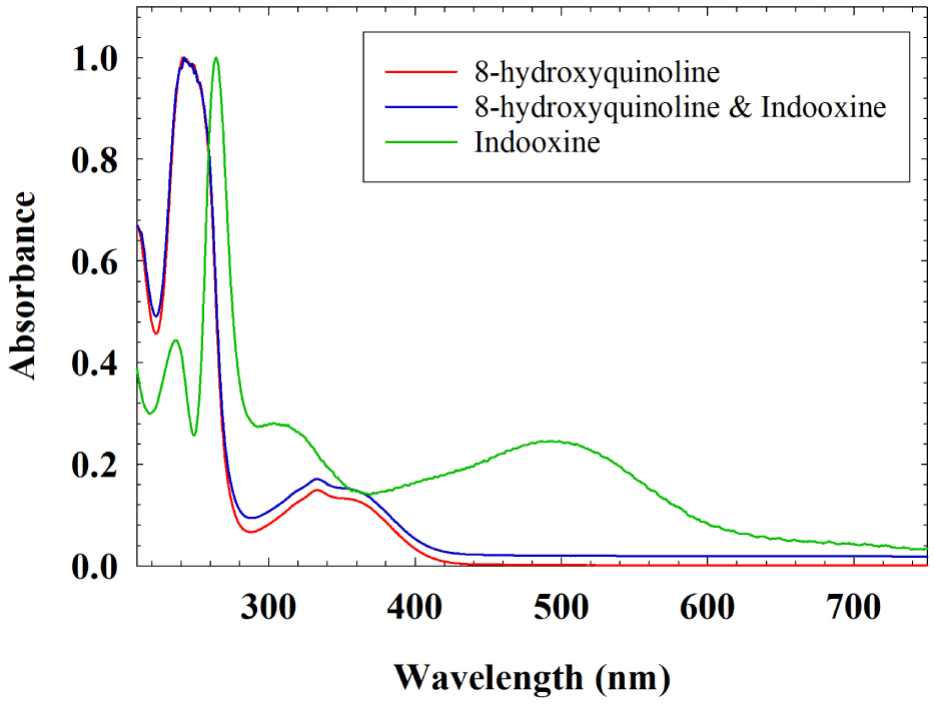
Normalized UV/vis absorbance spectra of solitary 8-hydroxyquinoline, 8-hydroxyquinoline with indooxine, and solitary indooxine.

The UV/vis absorption spectra demonstrates that shifting the wavelength from Frear and Burrell’s 705 nm to a UV wavelength allowed for a lower detection range. The indooxine absorption spectrum presented in green in Figure 1 was a sample of indooxine that was previously separated from 8-hydroxyquinoline and other reagents using the HPLC column prior to taking a measurement within the spectrophotometer. Indooxine’s peak absorptivity was found to occur at 261 nm, and the HPLC-UV detection wavelength was set accordingly. Numerous solvent ratio and gradient combinations for the HPLC were tested to separate the indooxine peak from the other reagent chemical absorbance peaks within the solution. The solvent ratio reported in the methods section was successful in isolating an indooxine peak, which was observed between 7 and 10 min into the sample run. The successful isolation of indooxine from other reagents, in a sample of an 8.0 μM indooxine solution with excess 8-hydroxyquinoline present is demonstrated in the chromatogram shown in Figure S1. It is likely that alternative mobile phase compositions or separation columns may achieve adequate distinction between the molecules, given that indooxine is approximately twice the size of 8-hydroxyquinoline.

To confirm the method’s sensitivity and a consistent, linear dose–response, a calibration curve was established using known NH_2_OH concentrations varying from 0 to 100 μM. Some instrumental drift was observed between dates; when measurements deviated beyond 1% of expected values, a new standard curve was prepared for a given sample set. A standard curve with concentrations consisting of 0.1, 1.0, 5.0, 10, 20, and 50 μM NH_2_OH were replicated three times to yield an averaged standard curve. A LoD for this NH_2_OH method was developed using the Clinical and Laboratory Standards Institute (CLSI)’s method for determining LoD.^20^ A 0.1 μM NH_2_OH solution was prepared then measured 21 times to determine the average concentration and standard deviation of the data point with the lowest concentration. These statistical parameters were used to determine the LoD for NH_2_OH with the HPLC method, according to the CLSI’s framework; the LoD for our method was found to be 0.05 μM NH_2_OH. Note that the presence of reagents needed to measure indooxine dilutes the NH_2_OH sample, so the indooxine standard curve values were adjusted to the concentration of NH_2_OH within the sample. See Figure S2 and Tables S3 and S4 for the standard curve and LoD data.

### Confirmation of Hydroxylamine Detection in Wastewater and River Water

After establishing the HPLC method for NH_2_OH detection in DI water, its performance was tested in more complex waters, such as wastewater and river water. Prior to preparing calibration curves in these samples, any existing NH_2_OH was removed by allowing it to decay according to our observations of NH_2_OH decay in neutral pH conditions, detailed below. The collected samples were kept at room temperature without acidification for at least 2 days before starting these experiments. After this dissipation period, the samples were then processed according to the standard protocols; they were acidified with H_2_SO_4_ to a pH below 2.0 and filtered prior to spiking with NH_2_OH. Three replicates of a calibration curve were prepared within wastewater and Mississippi River water samples spiked with known NH_2_OH concentrations. The results from both media types resulted in a repeatable standard curve, shown in Figure 2a, implying accurate recovery of NH_2_OH within these medias. Wastewater, Mississippi River water, and LSU lake water samples were all acidified to a pH below 2.0 using H_2_SO_4_ and tested to detect natural NH_2_OH concentrations within these samples, which ranged from 0.08 to 1.62 μM NH_2_OH, shown in Figure 2b.

**Figure 2:**
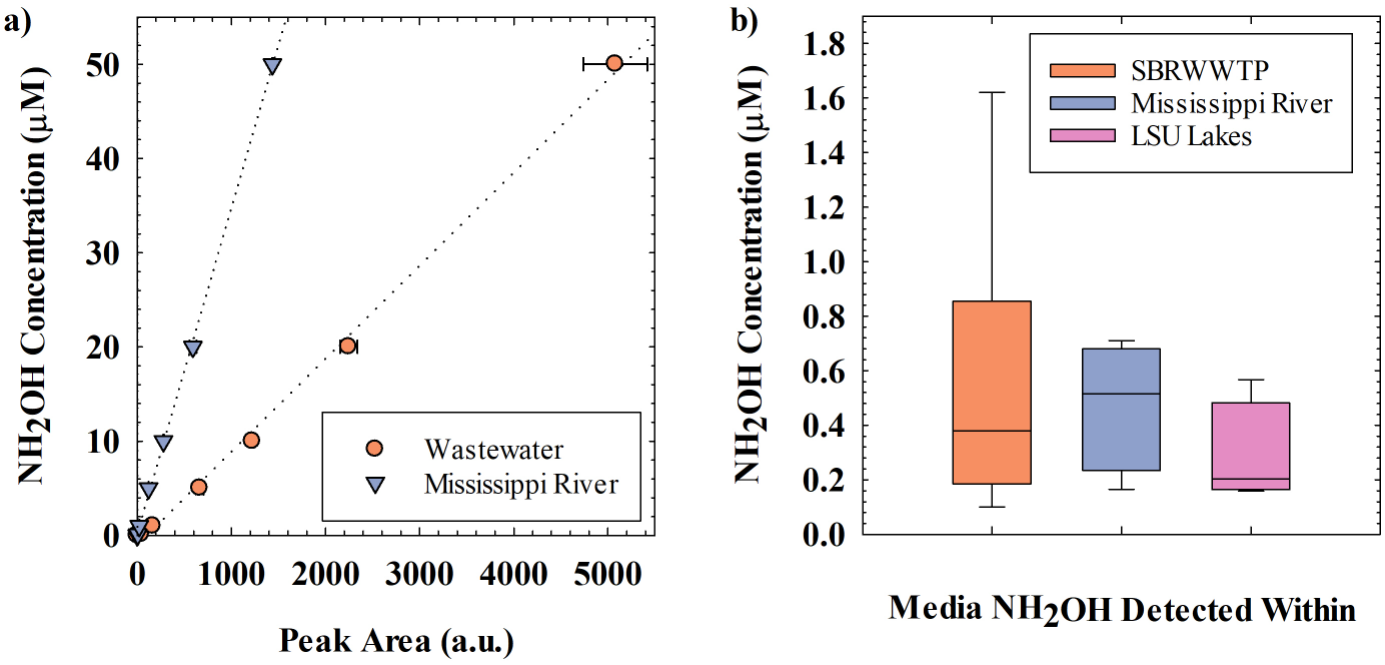
NH_2_OH detected in wastewater and river water at room temperature demonstrating a) calibration curves spiked with known NH_2_OH concentrations and b) raw NH_2_OH values in collected samples.

### Interference in River Water Samples

Precipitation occurred when Na_2_CO_3_ was added to the Mississippi River water samples and indooxine reagent mix (phosphate buffer, C_2_HCl_3_O_2_, and 8-hydroxyquinoline), so Frear and Burrell’s original method (20% NH_2_OH sample and 80% reagents)^16^ was used for all river and lake water samples. The formation of white precipitates (likely calcium carbonate) was not observed for river and lake water samples whenever using Frear and Burrell’s original recipe due to a higher dilution of the sample. Frear and Burrell’s recipe was tested using the same HPLC-UV method, which yielded a 0.10 μM LoD for NH_2_OH. The 20% NH_2_OH sample and 80% reagents method can be used for samples with constituents that interfere with the production of indooxine, such as a previously reported problem of forming precipitates within samples containing high concentrations of alkaline earth metal ions.^21^ In extreme cases, additional sample dilution may be necessary to avoid unwanted precipitation reactions. A higher dilution (i.e., 5% NH_2_OH sample and 95% reagents) was used to test brackish water samples and other water samples with high salinity contents; NH_2_OH detection with no precipitate interference was confirmed with this higher dilution ratio.

### Hydroxylamine Handling for Stability

The decay of NH_2_OH was tracked under varying pH conditions and temperatures to establish the most stable storage and handling conditions of the collected samples. The pH conditions used in these stability experiments included the following conditions at room temperature: an acidic solution below pH 2.0, a pH of 5.94, a neutral condition at pH 7, and a basic condition at pH 12, as shown in Figure 3a and 3b. An additional decay experiment was performed at NH_2_OH’s *p*Ka, which is 5.94.^22^ NaOH was used to raise the pH for the neutral and basic conditions, while HCl was used to lower the pH for acidic and *p*Ka pH conditions. A 0.05 M Na_2_HPO_4_ phosphate buffer set at a pH of 6.8 was used to stabilize the pH changes needed to reach a neutral pH.

**Figure 3:**
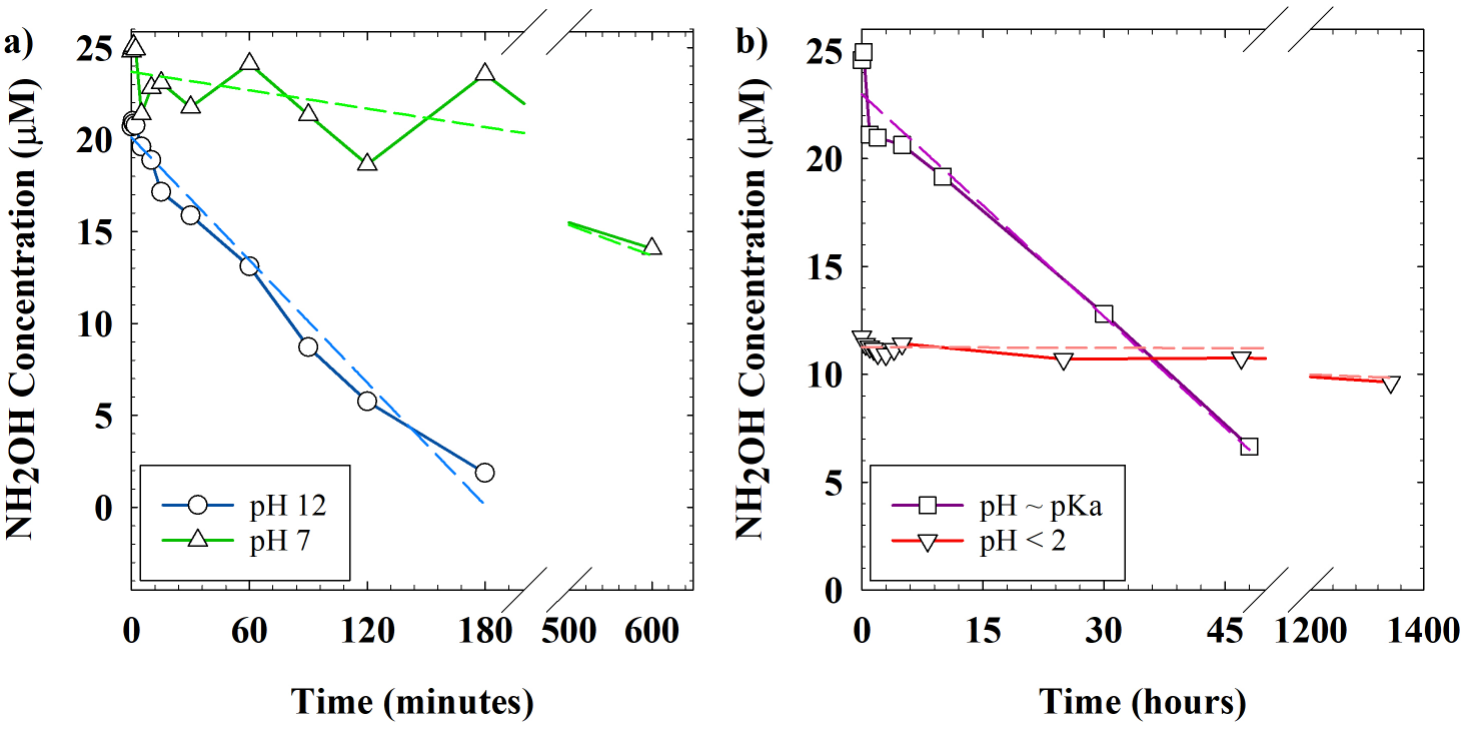
Room temperature decay of NH_2_OH at a) pHs 12 and 7 (shown in minutes) and b) at pH values set to the *p*Ka (5.94) and below 2.0 (shown in hours). Linear regression lines are shown as dashed lines.

The basic pH and neutral pH conditions had decay rate constants of −0.111 ± 0.006 and −0.016 ± 0.003, respectively, while the *p*Ka and acidic pH conditions had decay rate constants of −0.344 ± 0.029 and −0.001 ± 0.0002, respectively. NH_2_OH was much more stable under conditions of low pH such as the acidic and *p*Ka conditions. A solution of NH_2_OH with a pH below 2.0 remained at a relatively stable concentration for months. The pH conditions were tested for first-order decay, but the results agreed with a zeroth-order decay; the corresponding natural log plots are shown in Figure S3.

Two acids, HCl and H_2_SO_4_, were used to acidify the samples to a pH under 2.0 to test their respective ability to keep NH_2_OH stable. The results, shown in Figure 4, revealed that both acids kept NH_2_OH relatively stable for weeks at room temperature: up to two months before the NH_2_OH sample decayed by 10% of its starting concentration. The NH_2_OH sample acidified with HCl remained stable for several months before it notably decayed. These results suggest that HCl and H_2_SO_4_ can be used interchangeably for acidification of a nanopure water sample to a pH below 2.0 to prohibit the decay of NH_2_OH for two months at room temperature. The observation that NH_2_OH remained stable at conditions where the pH was below 2.0 confirmed that the acidification of samples is a sufficient measure to preserve them for analysis. The natural log plot of the decay of NH_2_OH at a pH under 2.0 using HCl versus H_2_SO_4_ for acidification is shown in Figure S4.

**Figure 4:**
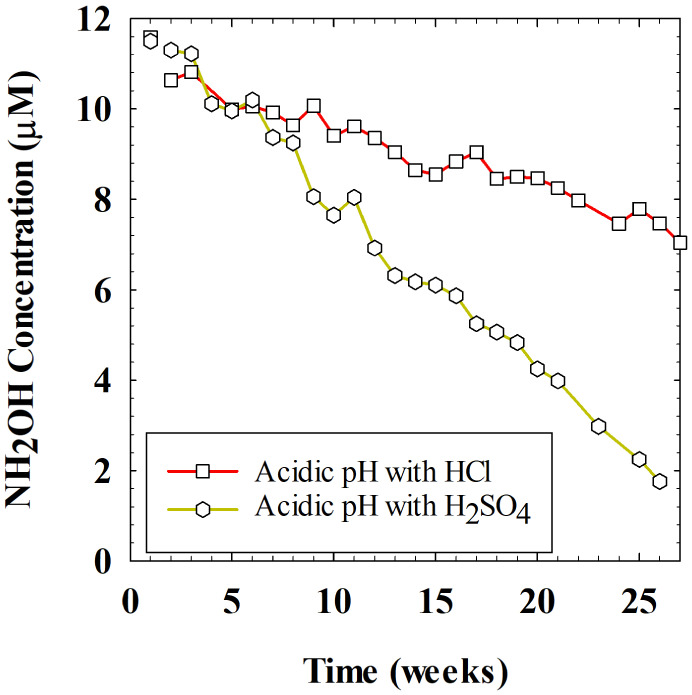
NH_2_OH decay in DI water acidified to a pH below 2.0 with either HCl or H_2_SO_4_ at room temperature.

Wastewater samples collected from the SBRWWTP and river water samples collected from the Mississippi River were spiked with NH_2_OH at a pH below 2.0 at room temperature. The decay of these samples was monitored, showing that a stable concentration of NH_2_OH was detected in both wastewater and river water for over a week from the collection date, as shown in Figure 5; the corresponding natural log plots are shown in Figure S5.

**Figure 5:**
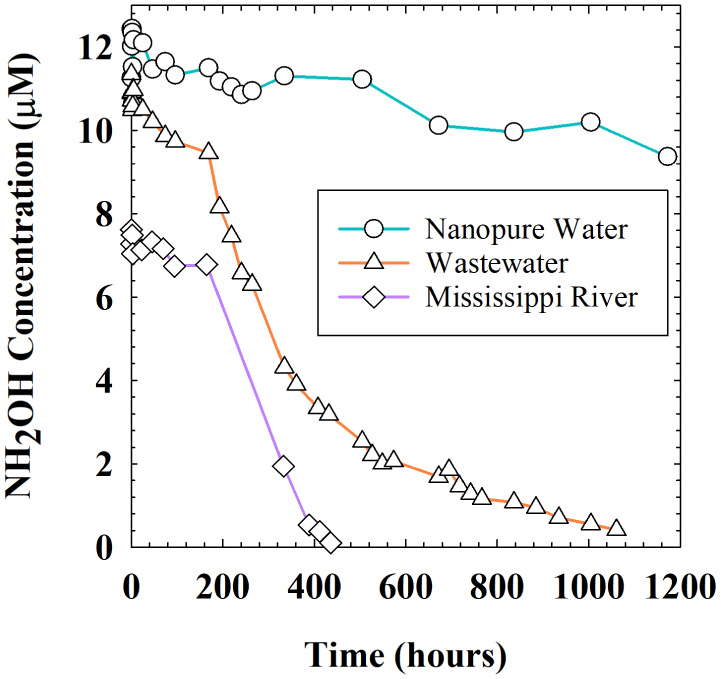
NH_2_OH stability in wastewater and river water at a pH below 2.0 at room temperature.

An additional decay experiment was performed to determine if the temperature of the NH_2_OH-containing sample influenced NH_2_OH decay kinetics. Nanopure water samples and wastewater samples at a pH below 2.0 were prepared and kept on the counter at room temperature and refrigerated. Decay was monitored daily for 1 week, but there was no observation of a difference in decay rates between the samples kept at room temperature versus refrigerated (data not shown). Notably, no readjustment of pH was needed prior to measuring the NH_2_OH concentration by reaction with 8-hydroxyquinoline to indooxine.

### Hydroxylamine’s Reactivity to Oxygen and Its Relationship with Stability

While it is well-known, and shown here in Figure 3, that NH_2_OH is not stable at neutral and basic conditions, the underlying decay reaction is not well understood. Fiadeiro et al. reported that deoxygenated solutions at higher pH values stayed relatively stable, similar to their oxygenated samples at a pH below 3.^3^ Here, we test if the presence of dissolved O_2_ causes NH_2_OH to decay under basic conditions. As shown in Figure 6, N_2_ gas was sparged into a sample containing a known concentration of NH_2_OH at a pH of about 12 at room temperature to purge all the dissolved oxygen out of the sample. The corresponding [NH_2_OH] versus time data is shown in Figure S6. After 2 h, the N_2_ gas was turned off and the dissolved oxygen was allowed to return to the NH_2_OH sample. Noticeably, while O_2_ was absent, NH_2_OH remained stable in the sample even under basic conditions. Once O_2_ was returned to the system, the NH_2_OH started to decay rapidly as the reaction returned to typical kinetics at a basic pH. It is important to note that under basic conditions (well above NH_2_OH’s *p*Ka), NH_2_OH will be present as NH_2_OH, whereas under acidic conditions, it will be present in its protonated form, NH_3_OH^+^. These observations indicate that NH_3_OH^+^ is not sensitive to O_2_, but NH_2_OH is reactive with O_2_, matching Fiadeiro et al.’s observations. Hydroxylamine is present primarily as NH_3_OH^+^ in acidic conditions, preventing the decay of NH_2_OH. While not tested here as a long-term solution, deoxygenation could be used as an alternative NH_2_OH preservation method in cases where acidification is not ideal.

**Figure 6:**
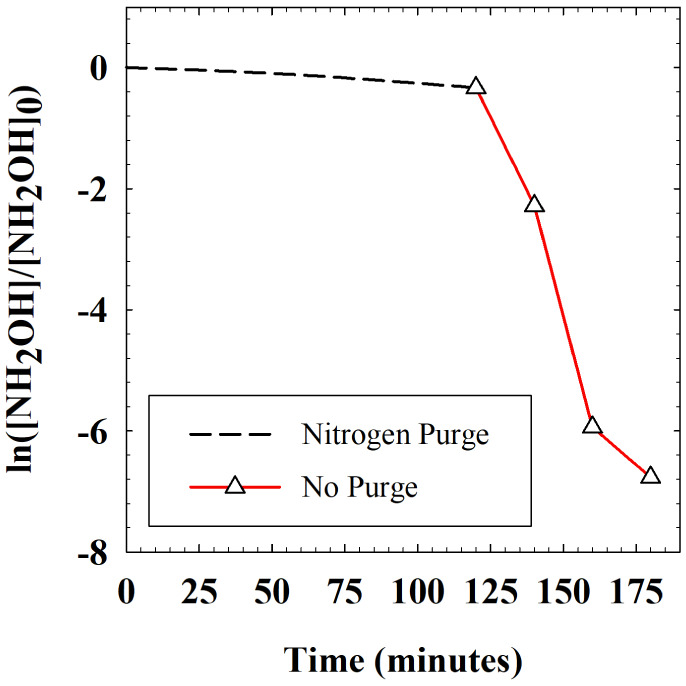
Natural log plot of NH_2_OH decay at pH 12 with and without N_2_ gas purging at room temperature.

## Conclusions

Our adaptation of this method allows for accurate quantification of NH_2_OH within low detection ranges, broadening the scope of NH_2_OH detection. We also demonstrate reliable environmental sampling procedures to capture stable measurements through the preservation of NH_2_OH. Our study of NH_2_OH decay kinetics reinforces that acidification of NH_2_OH samples below a pH of 2.0 is typically the best practice to ensure sample stability. The accurate and reliable detection of NH_2_OH opens pathways to understanding NH_2_OH’s role in nitrogen cycling. Detection of NH_2_OH was achieved in wastewater and river water with a vastly improved LoD, down to 0.05 μM NH_2_OH. We improved this method’s detection limit for many water samples, even when some river water cases needed Frear and Burrell’s original method to avoid precipitation interference. Alternately, researchers facing challenging waters may consider inducing precipitation through the addition of extra Na_2_CO_3_ to remove interfering components prior to analysis.

Enhanced NH_2_OH quantification in natural waters and wastewaters will improve our understanding of global nitrogen cycling. Soler-Jofra et al. noted that one of the primary limitations of understanding the role that NH_2_OH plays in nitrogen cycling is the lack of a convenient and nonproblematic method of NH_2_OH measurement.^1^ The presently described method provides an accessible procedure for NH_2_OH quantification using relatively common materials and equipment found in many environmental laboratories. Increasing sensitivity and accessibility of NH_2_OH determination will likely fuel the necessary research to shrink the knowledge gap of NH_2_OH as an intermediate compound within nitrification pathways in the global nitrogen budget. Furthermore, the sensitivity of this method may unlock a deeper understanding of nitrogen transformation pathways in wastewater, improving the prediction and understanding of N_2_O emissions from biological treatment systems.

## Supplementary Material



## Notes

Revised reagent recipe for the indooxine-NH_2_OH reaction, indooxine stability, NH_2_OH calibrations and calibration curves, HPLC chromatogram of isolated indooxine, and NH_2_OH decay controls (PDF)
