# Supplementary material for: Enhanced Environmental Hydroxylamine Detection: A Chromatographic Adaptation to the Indooxine Approach
Source: ACS Omega. 2026 Jul 8;11(28):42783–9. doi: 10.1021/acsomega.6c04322 (PMC13393357; doi:10.1021/acsomega.6c04322)
Supplement: Supplementary file 1 [file ao6c04322_si_001.pdf]

Supporting Information

Title: Enhanced Environmental Hydroxylamine Detection: A Chromatographic Adaptation to the Indooxine Approach

Authors: Madelyn Y. Hathcock Barden[1], Kevin M. McPeak[2], Samuel D. Snow[1*]

[1] Department of Civil and Environmental Engineering, 3255 Patrick Taylor Hall, Louisiana State University, Baton Rouge, Louisiana 70803
[2] Gordon and Mary Cain Department of Chemical Engineering, 3307 Patrick Taylor Hall, Louisiana State University, Baton Rouge, Louisiana 70803
*Corresponding author: ssnow@lsu.edu;

Contents
Number of pages: 5
Tables: 4
Figures: 6

Table S1. Revised reagent recipe for the indooxine-NH_2OH reactions solutions.

**Reagent**	**Stock Concentration (M)**	**Volume (mL)**
NH_2OH-containing sample	-	4.0
Phosphate buffer (6.8 pH)	0.4	0.125
Trichloroacetic acid	1.197	0.125
8-hydroxyquinoline	0.3	0.125
Sodium carbonate	1.0	0.625

Table S2. Stability of indooxine signal generated from a 100 µM NH_2OH sample after reaction with reagents (1 minute heating and 15 minutes cooling).

**Time after Cooling Period (Minutes)**	**Peak Area (a.u.)**
20	4,582.0
40	4,672.5
60	4,381.2
80	4,389.5
100	4,177.6
120	4,088.3
140	4,033.2

Table S3. NH_2OH concentrations used within the calibration curve with corresponding peak areas detected using the HPLC. Three replicates were performed for accurate detection.

**NH_2OH Concentration**	**Indooxine Concentration***	**Replicate 1 Peak Area (a.u.)**	**Replicate 2 Peak Area (a.u.)**	**Replicate 3 Peak Area (a.u.)**	**Average Peak Area (a.u.)**	**Standard Deviation**
0.00 µM	0.00 µM	0.0	0.0	0.0	0.0	0.0
0.10 µM	0.08 µM	20.9	17.2	17.2	18.4	2.1
1.00 µM	0.80 µM	177.2	206.6	278.4	220.7	52.0
5.00 µM	4.00 µM	997.7	977.3	1195.1	1056.7	120.3
10.0 µM	8.00 µM	2040.5	2145.2	2213.9	2133.2	87.3
20.0 µM	16.0 µM	3951.4	4328.6	4358.2	4212.7	226.8
50.0 µM	40.0 µM	9041.3	9727.8	10153.8	9640.9	561.3

*Note that the NH_2OH sample makes up 80% of the indooxine solution, diluted by the reagents comprising the remaining 20%.

Table S4. Replicates of 0.10 µM NH_2OH samples within the HPLC to determine the limit of detection (LoD). The 21 replicates resulted in an average peak area of 23.2 a.u. with a standard deviation of 6.1 a.u.

**Sample Run No.**	**Replicate 1 Peak Area (a.u.)**	**Replicate 2 Peak Area (a.u.)**	**Replicate 3 Peak Area (a.u.)**	**Replicate 4 Peak Area (a.u.)**	**Replicate 5 Peak Area (a.u.)**	**Replicate 6 Peak Area (a.u.)**	**Replicate 7 Peak Area (a.u.)**
1	20.9	18.5	27.0	26.2	23.4	24.5	22.7
2	17.2	29.8	20.6	28.6	31.9	31.0	29.9
3	17.2	35.4	24.1	32.1	36.3	35.0	34.4

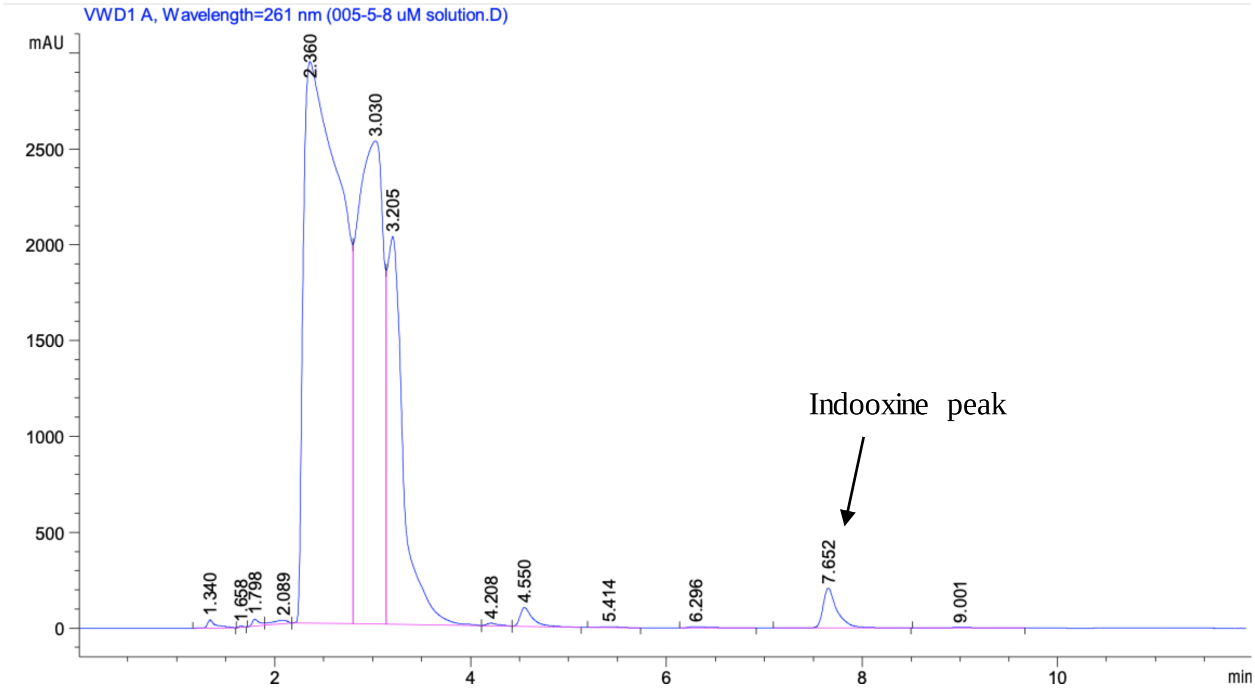

Figure S1. An HPLC chromatogram showing successful isolation of 8 µM indooxine (from a solution of 10 µM NH_2OH), with a peak at 7.65 min.

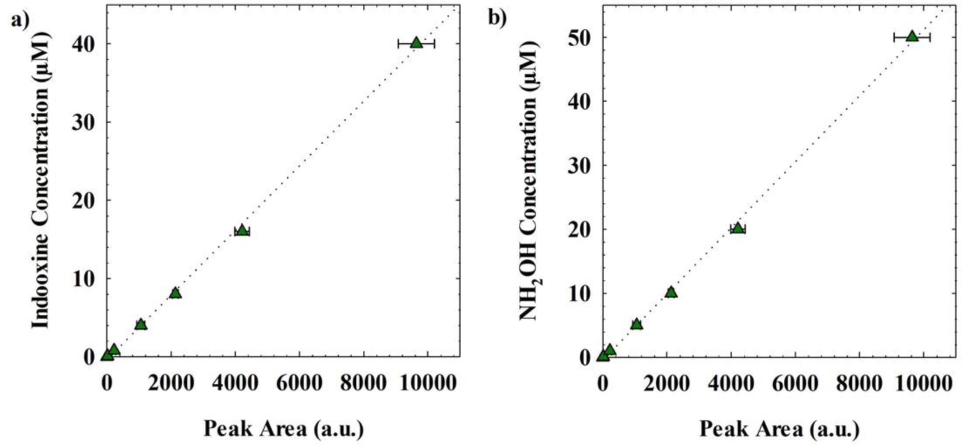

Figure S2. Calibration curve of a) 0-40 µM indooxine, corresponding to b) 0-50 µM NH_2OH, measured via HPLC.

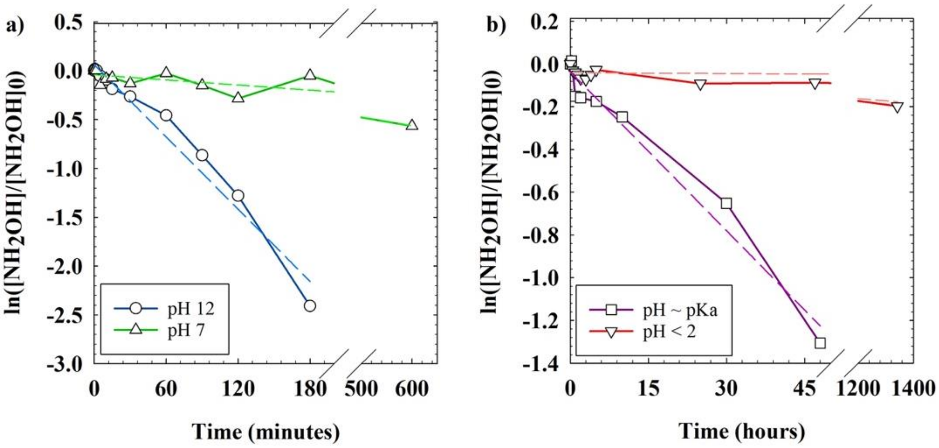

Figure S3. Natural log plots of room temperature decay of NH_2OH at pHs of a) approximately 12 and 7 (shown in minutes) and b) 5.94 (NH_2OH's *p*Ka) and below 2.0 (shown in hours). Linear regression lines are shown as the dashed lines corresponding to each experiment.

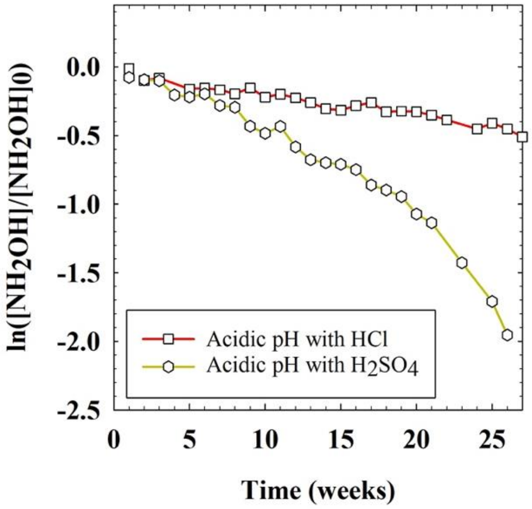

Figure S4. Natural log plot of NH_2OH decay in DI water acidified to a pH ≤ 2.0 with either HCl or H_2SO_4 at room temperature.

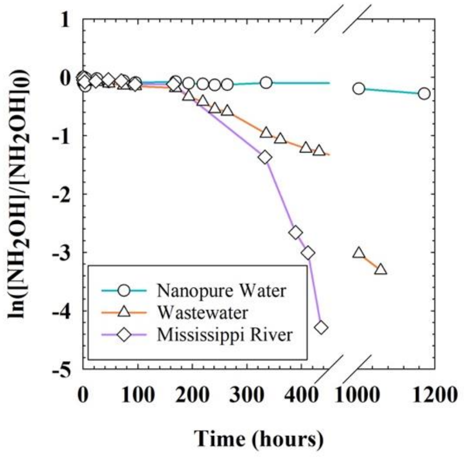

Figure S5. Natural log plot of NH_2OH decay in wastewater and river water at pH ≤ 2.0 at room temperature.

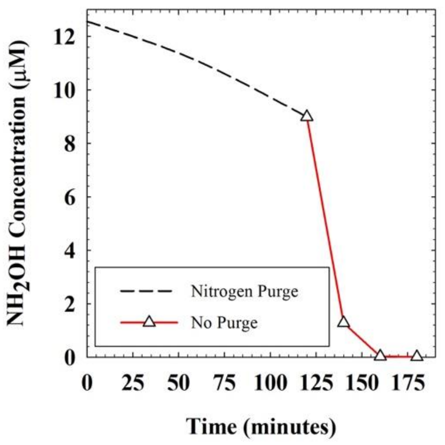

Figure S6. NH_2OH decay at pH 12 with and without N_2 gas purging at room temperature.
